# Temporal and spatial changes in reactive astrogliosis examined by ^18^F-THK5351 positron emission tomography in a patient with severe traumatic brain injury

**DOI:** 10.1186/s41824-021-00121-2

**Published:** 2021-12-23

**Authors:** Tetsuhiro Hatakeyama, Kenya Kawakita, Nobuyuki Kawai, Hajime Shishido, Yuka Yamamoto, Yoshihiro Nishiyama, Keisuke Miyake

**Affiliations:** 1 Department of Neurological Surgery, Faculty of Medicine, Kagawa University, Miki-cho, Kagawa Japan; 2 Department of Emergency and Critical Care Medicine, Kagawa University Hospital, Miki-cho, Kagawa Japan; 3 Department of Neurological Surgery, Kagawa Rehabilitation Hospital, 1114 Tamura-cho, Takamatsu-shi, Kagawa 761-8057 Japan; 4 Department of Diagnostic Radiology, Faculty of Medicine, Kagawa University, Miki-cho, Kagawa Japan

**Keywords:** ^18^F-THK5351, Traumatic brain injury, Reactive astrogliosis, Positron emission tomography

## Abstract

**Background:**

The positron emission tomography (PET) radioligand ^18^F-THK5351 is now used to evaluate monoamine oxidase B expression in the reactive astrogliosis seen in various central nervous diseases. Traumatic brain injury (TBI) is known to induce reactive astrogliosis in the lesion site. This is a first report to examine the spatial and temporal changes in reactive astrogliosis as evaluated by ^18^F-THK5351 after a severe TBI.

**Case presentation:**

A 27-year-old man suffering from a severe TBI with multiple brain contusions was examined using ^18^F-THK5351 PET/CT in the subacute and chronic phases after the injury. The first PET scan, performed 46 days after the TBI, showed intense uptake of ^18^F-THK5351 in and around the brain contusions. The second PET scan, performed 271 days after the TBI, showed reduced uptake of ^18^F-THK5351 at the original sites of the brain contusions and increased uptakes in the white matter surrounding the contusions and the corpus callosum. The patient exhibited sustained improvement of neuropsychological impairment between the two PET examinations and remarkable recovery from the severe TBI.

**Conclusions:**

There were evident temporal and spatial changes in ^18^F-THK5351 uptake in the traumatized brain between the two PET examinations. These changes may have been related to the remarkable neurological recovery in this patient. The degree and distribution of reactive astrogliosis detected by ^18^F-THK5351 PET may be useful in assessing pathophysiology and predicting prognosis in TBI patients.

## Background

The positron emission tomography (PET) radioligand ^18^F-THK5351 was developed for imaging tau aggregates in neurofibrillary tangles (NFTs) in patients suffering from Alzheimer disease (AD; Harada et al. 2016). However, ^18^F-THK5351 is retained in the basal ganglia and other brain regions known to accumulate trace deposits of NFTs in AD, which raises concerns about the specificity of ^18^F-THK5351 for imaging tau aggregates. A later study demonstrated a substantial reduction of ^18^F-THK5351 uptake in the brain after a single administration of the monoamine oxidase B (MAO-B) inhibitor, selegiline (Ng et al. 2017). Furthermore, in a study comparing antemortem ^18^F-THK5351 PET images and postmortem pathological specimens in a patient with AD, in vivo ^18^F-THK5351 uptakes were significantly correlated with both the density of tau aggregates in the neocortex and regional MAO-B concentrations in the whole brain (Harada et al. 2018). According to these studies, it is now recognized that ^18^F-THK5351 is a dual-purpose compound that binds to both tau aggregates and MAO-B. In cases in which tau pathology is particularly irrelevant, such as young subjects in whom tau protein does not usually accumulate, ^18^F-THK5351 uptake is mainly considered to reflect the MAO-B concentration.

Traumatic brain injury (TBI) induces increased astrogliosis in and around the lesion site (Burda et al. 2016), and reactive astrocytes overexpress MAO-B in the outer mitochondrial membrane (Levitt et al. 1982). Recently, Takami et al. reported a case, in which ^18^F-THK5351 PET could identify lesions based on the reactive astrogliosis in a brain contusion 18 days after TBI (Takami et al. 2020). However, their PET study was a snapshot. More importantly, temporal and spatial assessment of ^18^F-THK5351 uptake can provide new insights into the pathophysiology of TBI in relation to the role of reactive astrogliosis.

Here, we report a young patient suffering from a severe TBI, in which the degree and distribution of reactive astrogliosis were examined by ^18^F-THK5351 PET in the subacute and chronic phases after TBI. This is the first study to examine the temporal and spatial changes in ^18^F-THK5351 uptake in a patient with a severe TBI. The findings may suggest different roles of reactive astrogliosis in the subacute and chronic phases after TBI, which could be detected by ^18^F-THK5351 PET in the human brain.

## Case presentation

**Fig. 1:**
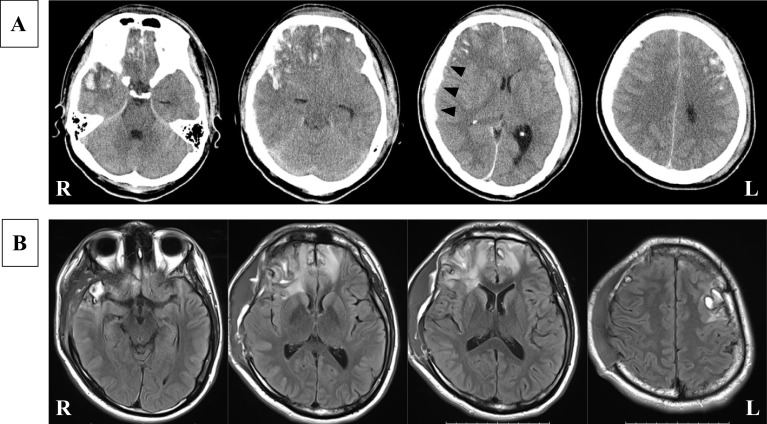
**A** Head CT scan on the day of injury showed multiple brain contusions (salt and paper appearance) in the right frontal and temporal lobes and left frontal lobe along with a right acute subdural hematoma (arrow heads). **B** FLAIR MR imaging 23 days after the injury showed multiple high-intensity regions in the right frontal and temporal lobes and left frontal lobe along with high-intensity areas in the white matter surrounding the brain contusions

The patient was a 27-year-old man without significant medical history. He had high level of education and worked as a veterinarian. He had been extremely intoxicated one night and had been found the next morning in a ditch in a disturbed state of consciousness. He was transported to an emergency hospital, and a head CT scan showed brain contusions in the right frontal and temporal lobes and left frontal lobe, with a right acute subdural hematoma (Fig. 1A). He was referred to the Emergency Medical Center of Kagawa University Hospital. On admission, his consciousness level was GCS 7 (E1V1M5). A catheter was inserted to monitor his intracranial pressure (ICP) and his initial ICP was 38 mmHg under deep sedation and administration of hyperosmotic solution. He subsequently underwent an emergency right frontotemporoparietal craniectomy for evacuation of the acute subdural hematoma. Targeted temperature management (TTM) with a target temperature of 36ºC was conducted as a neuroprotective therapy and continued for several days under close monitoring of the ICP. He gradually regained consciousness after the discontinuation of sedatives for the TTM. He was mostly alert, with some memory confusion and retrograde amnesia, about 20 days after the TBI. Fluid-attenuated inversion recovery MR imaging 23 days after the TBI showed multiple high-intensity regions in the right frontal and temporal lobes and left frontal lobe, with high-intensity areas in the white matter surrounding the brain contusions (Fig. 1B).

The clinical use of ^18^F-THK5351 tracer was approved by the Kagawa University Faculty of Medicine Human Subjects Ethical Committees, and informed consent was obtained from the patient, with written consent received before the PET examinations. The ^18^F-THK5351 PET examinations were performed using a Biograph mCT PET/CT scanner (Siemens Medical Solutions USA Inc., Knoxville, TN, USA). Attenuation correction was performed using CT maps. Regional emission scans were acquired for 20 min, beginning 50 min after an intravenous bolus injection of 3.7 MBq/Kg of ^18^F-THK5351. The PET data were acquired in three-dimensional mode and were reconstructed with the baseline ordered-subsets expectation maximization algorithm. The ^18^F-THK5351 PET images were then normalized using the cerebellum as a reference region, with cerebellar uptake set as one.

**Fig. 2:**
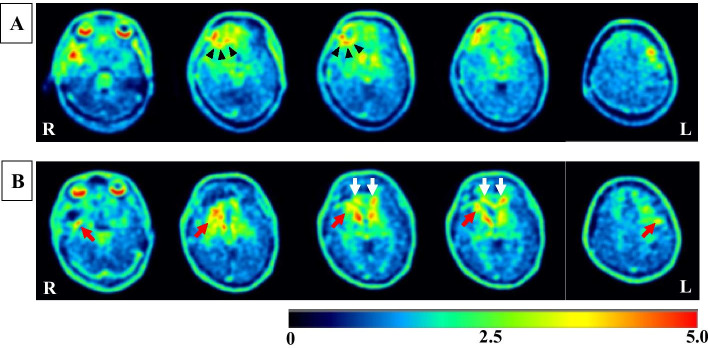
^18^F-THK5351 PET images 46 days (**A**) and 271 days (**B**) after the injury. The rainbow color bar represents the normalized uptake of ^18^F-THK5351, with cerebellar uptake set as one. **A** PET images showed increased uptake of ^18^F-THK5351 in the right frontal and temporal lobes and in the left frontal lobe and increased ^18^F-THK5351 uptake with a ring-like pattern around the brain contusion in the right frontal lobe (black arrow heads). **B** PET images showed reduced uptake of ^18^F-THK5351 in the original sites of brain contusions in the right frontal and temporal lobes and in the left frontal lobe. Increased ^18^F-THK5351 uptakes were observed in the white matter surrounding the brain contusions (red arrows) and the corpus callosum (white arrows)

The images from his First ^18^F-THK5351 PET scan, which was performed 46 days after the TBI, revealed high uptake of ^18^F-THK5351 in the right frontal and temporal lobes and in the left frontal lobe (Fig. 2A). Increased ^18^F-THK5351 uptake was noted in a ring-like pattern around the brain contusion in the right frontal lobe (Fig. 2A). He underwent autologous cranioplasty on day 33 and was referred to a rehabilitation hospital to receive in-hospital rehabilitation for neuropsychological impairment 51 days after the TBI. The rehabilitation program was directed to improve attention, memory, visuospatial abilities, and executive functioning. He recovered well after cognitive rehabilitation for a month, with high scores in neuropsychological examinations. He was discharged to home on day 82, and he returned to the work as a veterinarian about 4 months after the TBI and had no difficulty in working. The images from his second ^18^F-THK5351 PET scan, which was performed 271 days after the TBI, showed reduced uptake of ^18^F-THK5351 in the original sites of the brain contusions in the right frontal and temporal lobes and in the left frontal lobe (Fig. 2B). At that time, increased ^18^F-THK5351 uptakes were observed in the white matter surrounding the brain contusions and the corpus callosum (Fig. 2B).

## Discussions

The radioligand ^18^F-THK5351 was developed for imaging tau aggregates in NFTs in AD (Harada et al. 2016). Repetitive mild TBI can trigger the development of chronic traumatic encephalopathy (CTE), a progressive neurodegeneration characterized by the widespread deposition of hyperphosphorylated tau proteins as NFTs (McKee et al. 2009, 2013). In professional sports, there is increasing concern that football players and boxers with a history of repeated mild TBI might have an increased risk of developing CTE (McKee et al. 2009). There is a long latency period, usually 8–10 years, between an individual’s exposure to repetitive TBI and the appearance of clinical symptoms and tau aggregates in CTE (McKee et al. 2009). Therefore, the changes in ^18^F-THK5351 uptake in the present case are unlikely to be associated with tau aggregates in the injured brain after a single TBI.

TBI is characterized by initial brain damage caused by direct mechanical force and secondary damage due to subsequent pathophysiological processes. Following the initial injury, local microenvironment changes and damaged cells release intracellular components, triggering the activation and recruitment of resident glial cells in the injured brain. Among the resident glial cells in the brain, astrocytes are ubiquitous cells throughout brain tissue and play crucial roles in maintaining the homeostasis of ions, transmitters, water, and blood flow that are crucial for neuronal function in the brain (Chen and Swanson 2003). Astrocytes are pivotal responders to injury through diverse potential changes commonly referred to as reactive astrogliosis (Pekny and Nilsson 2005). TBI induces reactive astrocytes in and around the lesion site and is accompanied by a prominent increase in MAO-B expressions in reactive astrogliosis (Burda et al. 2016; Levitt et al. 1982). Both beneficial and detrimental roles have been attributed to reactive astrogliosis after TBI onset. For example, a potential tissue-protective effect could be provided by glutamate uptake, free radical scavenging or neurotrophin release, while potentially harmful effects might be caused by the release of pro-inflammatory cytokines or cytotoxic radicals (Chen and Swanson 2003). In the present case, ^18^F-THK5351 PET 46 days after the TBI showed a ring-like pattern tracer uptake around the brain contusions, suggesting that glial scar formation isolated the damaged area and restricted the spread of inflammatory cells and compounds, which would provide a favorable environment for surviving neurons.

At 271 days after the TBI, ^18^F-THK5351 PET showed reduced uptake of ^18^F-THK5351 in the original sites of brain contusions and increased tracer uptake in the white matter surrounding the brain contusions and the corpus callosum. A recent animal study showed that a single severe TBI resulted in a long-term, progressive inflammatory process with activated microglia and astrocytes (Pischiutta et al. 2018). This process not only involved the peri-lesional and ipsilateral hemisphere, but also extended to the contralateral hemisphere one year after injury, with particular involvement of white matter structures including the corpus callosum (Pischiutta et al. 2018). Increased uptake of ^18^F-THK5351 within the corpus callosum in the chronic phase is of particular interest, as this structure represents a region highly susceptible to TBI, suggesting that active neuroinflammatory events driven by reactive astrogliosis are continued at this time. This finding reflects clinical observations in moderate to severe TBI patients: an ongoing neuroinflammatory response was evident in the corpus callosum in about one-third of the patients surviving a year or more after a single TBI (Johnson et al. 2013). There are increasing evidence that reactive astrocytes also play crucial roles in post-TBI synaptic plasticity and the reorganization of the neuronal circuits (Burda et al. 2016). Astrocytes have a dual (promoting or suppressing) role in neuronal plasticity and reconstruction, including neurogenesis, synaptogenesis, angiogenesis, and repair of the blood–brain barrier after TBI (Zhou et al. 2020). The overall effects of sustained reactive astrogliosis after TBI have not been fully elucidated and may vary with injury intensity, distance from lesions, and stage of injury. Other cells such as brain-resident microglia are also activated after TBI. Both astrocytes and microglia react within 24 h and peak around day 3–7; however, microglia rapidly decline to control levels approximately 21 days, while astrocytes exhibited a long-lasting activation, at least, 28 days after TBI (Jassam et al. 2017). Studies have shown that the 18 kDa translocator protein (TSPO) expression is upregulated in activated microglia cells in response to inflammation or injury to the brain and many radioligands have been synthesized for TSPO imaging (Tronel et al. 2017). A multi-targets approach of PET imaging for astrocytes and microglia activation can help to characterize the involvement of neuroinflammation after TBI.

Recently, Ishibashi et al. reported a case of cerebral infarction in which astrogliosis in the lesion was monitored by ^18^F-THK5351 PET on days 27 and 391. The uptake of ^18^F-THK5351 in the infarct lesion decreased significantly between the two PET examinations (Ishibashi et al. 2020). The change in ^18^F-THK5351 uptake in the infarct lesion was different from our case, suggesting that different brain pathologies induce different spatial and temporal changes in reactive astrogliosis.

## Conclusion

In the present case report, we firstly evaluated the temporal and spatial changes in reactive astrogliosis using ^18^F-THK5351 PET after a severe TBI. The results showed dynamic changes in ^18^F-THK5351 uptakes in and around the lesions between days 46 and 271, along with sustained improvement of neuropsychological impairment. Intense ^18^F-THK5351 uptake in and around the damaged areas may suggest glial scar formation for surviving neurons in the early phase and neuronal plasticity and reconstruction in the chronic phase, as the patient exhibited remarkable recovery from the severe TBI. These findings suggest that the temporal and spatial changes in ^18^F-THK5351 uptake may reflect different functions of reactive astrogliosis after TBI. Further study of ^18^F-THK5351 PET with more cases can clarify the specific roles of reactive astrogliosis and is useful in assessing pathophysiology and predicting prognosis in TBI patients.

## Data Availability

The datasets used and/or analyzed in the current study are available from the corresponding author on reasonable request.

## Abbreviations

PET: Positron emission tomography
NFTs: Neurofibrillary tangles
AD: Alzheimer disease
MAO-B: Monoamine oxidase B
TBI: Traumatic brain injury
ICP: Intracranial pressure
TSPO: 18 KDa translocator protein
TTM: Targeted temperature management
CTE: Chronic traumatic encephalopathy
